# 3D Printing of PVA Capsular Devices for Applications in Compounding Pharmacy: Stability Evaluation and In Vivo Performance

**DOI:** 10.3390/pharmaceutics17050613

**Published:** 2025-05-05

**Authors:** Juan Francisco Peña, Daniel Andrés Real, Juan Pablo Real, Santiago Daniel Palma, María del Pilar Zarazaga, Nicolás Javier Litterio, Loreana Gallo, Ivana Maria Cotabarren

**Affiliations:** 1 Planta Piloto de Ingeniería Química, PLAPIQUI (UNS-CONICET), Camino La Carrindanga Km 7, Bahía Blanca 8000, Argentina; jfpena@plapiqui.edu.ar (J.F.P.); lgallo@plapiqui.edu.ar (L.G.); 2 Departamento de Ingeniería Química, Universidad Nacional del Sur (UNS), Av. Alem 1253, Bahía Blanca 8000, Argentina; 3 Unidad de Investigación y Desarrollo en Tecnología Farmacéutica, Consejo Nacional de Investigaciones Científicas y Tecnológicas, Facultad de Ciencias Químicas, Universidad Nacional de Córdoba, Av. Haya de la Torre y Medina Allende, Córdoba 5000, Argentina; real.daniel.andres@gmail.com (D.A.R.); juan.real@unc.edu.ar (J.P.R.); sdpalma@unc.edu.ar (S.D.P.); 4 Pill.AR Apotheke Revolution S.A, Córdoba 5000, Argentina; 5 Facultad de Ciencias Agropecuarias, IRNASUS CONICET, Universidad Católica de Córdoba, Córdoba 5016, Argentina; pzarazaga@ucc.edu.ar (M.d.P.Z.); nlitterio@ucc.edu.ar (N.J.L.); 6 Departamento de Biología, Bioquímica y Farmacia, Universidad Nacional del Sur (UNS), San Juan 670, Bahía Blanca 8000, Argentina

**Keywords:** magistral compounding, 3D printing, fused deposition modeling, in vivo studies, capsular devices, stability studies

## Abstract

**Background**: The personalization of medication through 3D printing enables the development of capsular devices (CDs) tailored to patient-specific needs. This study aimed to evaluate the stability and in vivo performance of 3D-printed polyvinyl alcohol (PVA) CDs with 0.4 and 0.9 mm width wall thicknesses (WT) compared to traditional hard gelatin capsules (HGCs). **Methods**: Capsules were tested for swelling, erosion, adhesion, water sorption, and in vitro disintegration. Additionally, the release of the model drug (losartan potassium) from CDs was evaluated. In vivo capsule opening times were assessed in dogs using X-ray imaging. Stability studies were conducted under natural (25 ± 2 °C, 60 ± 5% RH) and accelerated (40 ± 2 °C, 75 ± 5% RH) storage conditions. **Results**: CDs with 0.4 mm WT (CD–0–0.4) exhibited higher swelling and erosion, lower adhesion, and faster disintegration, leading to a more immediate drug release, comparable to HGCs. A strong correlation was found between in vitro and in vivo disintegration behavior. Water sorption tests revealed lower moisture affinity for PVA CDs compared to HGC. Stability studies showed that CD–0–0.4 retained its physical and chemical properties. Instead, CDs with 0.9 mm WT (CD–0–0.9) were sensitive to storage, particularly under accelerated aging, which affected their integrity and release profile. **Conclusions**: These findings highlight the potential of PVA-CDs, especially the 0.4 mm design, as a promising and stable alternative for compounding pharmacy applications, offering an effective platform for personalized oral drug delivery.

## 1. Introduction

The personalization of medication through 3D printing presents great opportunities for compounding pharmacy applications [1,2]. This innovative technology facilitates the design and production of capsular devices (CDs) specifically customized to meet the unique needs of individual patients, addressing variations in dosage, release profiles, and even the combination of multiple drugs within a single device [3]. For such applications, polyvinyl alcohol (PVA) is a widely adopted polymer in pharmaceutical 3D printing due to its biocompatibility. In addition, the advantageous mechanical properties of PVA support the fabrication of robust and structurally stable forms. Moreover, its water solubility enables precise control over drug release [4,5]. Recently, Peña et al. [6] designed and fabricated 3D-FDM-printed PVA-CDs with various wall thicknesses (WTs) and sizes to be adopted in pharmaceutical compounding. The CDs were printed and filled with a model antihypertensive drug (losartan potassium) within the same procedure. The CDs showed distinct release profiles based on WT. A 0.9 mm WT enabled modified (delayed) drug release, while a 0.4 mm WT achieved immediate drug delivery, mirroring traditional hard gelatin capsules’ (HGCs) performance. While traditional HGCs are typically produced in standardized, large-scale batches by the pharmaceutical industry, the use of 3D printing introduces a new paradigm, enabling the on-demand fabrication of customized CDs within the compounding pharmacy. In vitro characterization of these CDs highlighted the potential for their use, showing promising drug release behaviors. However, in vivo studies are essential to gain a comprehensive understanding of their behavior within the body and to account for the complex interactions that occur in a biological environment [7]. In this sense, Genina et al. [8] performed in vivo pharmacokinetic studies in rats to evaluate the performance of dual-compartment dosage units printed in PLA and PVA for administering rifampicin and isoniazid. The results showed slower and more prolonged drug release and absorption compared to drug-loaded free filaments. Goyanes et al. [9] used positron emission tomography/computed tomography in rats to track the movement and disintegration of 3D-printed CDs made with different polymers (PVA-PEG, HPC, HPMCAS). Results showed that devices manufactured with PVA-PEG copolymer disintegrated after 60 min. Smith et al. [10] evaluated the in vivo drug release of 3D-printed PVA capsules in beagle dogs and compared the plasma concentration profiles with an immediate-release tablet formulation. The results demonstrated that the 3D-printed capsules provided distinct release rates. Charoenying et al. [11] performed in vivo studies in rabbits using a floating, assemblable CD printed via FDM with PVA and loaded with barium sulfate. Through X-ray imaging, they demonstrated the device’s ability to float in the stomach for over 10 h. Kotha et al. [12] conducted an in vivo pharmacokinetic study in rabbits to observe the release pattern of a hollow, assemblable CD printed using Kollicoat MAE 100P filament. The study demonstrated a delay in drug release, confirming the enteric release behavior of the device. Seoane-Viaño et al. [7] recently explored the correlation between in vivo and in vitro disintegration time of 3D-printed tablets (torus shape) produced by selective laser sintering printing technology. The study compared the standard disintegration method of the United States Pharmacopoeia with an alternative method conducted in petri dishes. The latter method led to a better correlation. However, no clear in vivo/in vitro correlation was found, emphasizing the need to develop a more reliable in vitro methodology for 3D-printed dosage forms. Both HGC and PVA-CDs play a fundamental role in protecting the drug, ensuring its effectiveness until it reaches the site of absorption in the body. The ability of these devices to safeguard the drug from environmental factors (e.g., moisture, temperature) is essential for maintaining efficacy over time. Long-term stability studies of 3D-printed CDs are therefore of paramount importance. These tests assess not only how well the CD preserves the drug over time but also how the CD itself withstands various storage conditions. Understanding the stability of these CDs allows pharmacists to predict shelf life, optimize storage protocols, and offer patients more reliable and effective treatments. Notably, there is a lack of published literature on the stability of 3D-printed pharmaceutical forms, highlighting the need for additional research in this area to ensure the long-term efficacy and safety of these personalized medicines [13]. In fact, it is necessary to refer to general oral solid dosage forms and various 3D printing technologies to encounter stability tests. Recently, Rodríguez-Maciñeiras et al. [14] conducted a comparative stability study of capsules filled with a 3D-printed ink containing minoxidil and capsules manufactured using traditional methods. All the capsules remained stable over the period studied (90 days). On the other hand, Denis et al. [15] developed combined dosage units with tamoxifen and antidepressants using a modified FDM printer to dispense the solid drugs into commercial HGC, confirming the drug’s stability for up to one year at room temperature. Goyanes et al. [16] utilized semi-solid extrusion to produce chewable isoleucine tablets and demonstrated their stability for one month under accelerated conditions. Gültekin et al. [17] manufactured immediate-release pramipexole tablets using FDM and conducted long-term (twelve months) and accelerated (six months) stability tests, finding that the optimal formulation was stable under both conditions. Keikhosravi et al. [18] printed polypills containing aspirin and simvastatin via FDM, confirming their physical and drug content stability for two months under accelerated conditions. Pereira et al. [19] designed PLA and PVA capsules with parallel and concentric designs for controlled drug release, demonstrating stability during processing and one month of accelerated storage. Finally, Vakili et al. [20] printed propranolol doses on edible substrates using thermal inkjet printing, observing immediate drug release and stability for three months, with some drug recrystallization on starch-based substrates.

In this context, the novelty of this work lies in providing a comprehensive in vivo and in vitro evaluation of the pharmaceutical performance and storage stability of 3D-printed CDs, which is still scarcely reported in the literature. These findings offer key insights into the robustness and applicability of PVA-based CDs for use in compounding pharmacy applications, supporting their potential as a stable, customizable platform for oral drug delivery. To this end, we examined FDM-printed CDs with WTs of 0.4 mm and 0.9 mm, comparing them with HGCs. Building upon our previous work, these specific designs were selected due to their distinct drug release profiles. Swelling/erosion, adhesion, and water sorption of the CDs were evaluated. Stability was tested under natural (25 ± 2 °C, 60 ± 5% RH) and accelerated (40 ± 2 °C, 75 ± 5% RH) storage conditions over one and three months. Key quality attributes, such as weight, dimensions, disintegration time, and in vitro dissolution profiles, were evaluated and compared to time zero. Additionally, the model drug’s chemical nature and structure were evaluated after storage. Moreover, the in vivo performance of these devices was evaluated in dogs, focusing on the in vivo disintegration time of CDs and HGC compared to in vitro results. To summarize, this work uniquely combines formulation design, in vivo validation, and regulatory-relevant stability testing of 3D-printed PVA-based CDs. This multi-angle evaluation contributes valuable evidence supporting their applicability in patient-specific therapies.

## 2. Materials and Methods

### 2.1. Materials

The CDs were manufactured using a 1.75 mm diameter polyvinyl alcohol (PVA) filament (e-SUN, Shenzhen, China). The CDs were filled with losartan potassium (LP, 99.8% purity over anhydrous basis). For in vivo analyses, the CDs were filled with barium sulfate (BaSO_4_, Cicarelli, Santa Fe, Argentina) as a contrast agent. The background spectra of the Fourier Transform Infrared (FT-IR) spectroscopy technique were collected using spectroscopic-grade potassium bromide (KBr, Merck, Darmstadt, Germany).

### 2.2. Capsular Devices Design and 3D Printing Process

The CDs were printed on a Prusa-type FDM printer (Prusa I3 Hephestos, Buenos Aires, Argentina) using the same parameters, raw materials, and printing steps as Peña et al. [6]. The CDs were loaded with 500 mg of LP.

**Figure 1:**
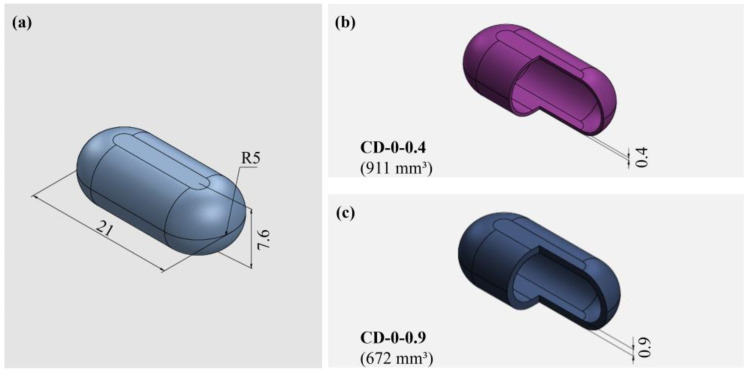
CAD designs of CDs: (**a**) isometric view, (**b**), (**c**) internal views of CDs 0–0.4 and 0–0.9, respectively.

For this analysis, CDs with an internal volume equivalent to that of a size 0 HGC (670 mm^3^) with WTs of 0.4 mm (CD–0–0.4) and 0.9 mm (CD–0–0.9) were selected (Figure 1).

To ensure reproducibility, the printing conditions were consistent with our previous work [6]. The CDs were printed in two stages to allow manual filling with LP. The body (layers 1–63) was printed at 190 °C with a flow of 100%. The cover (layers 64–75) was printed at 200 °C, with flow increasing to 170% for layers 64–69 and 150% for layers 70–75. Printing speed changed from 1200 mm/min to 250 mm/min for layers 64–66, 100 mm/min for layer 67, and back to 250 mm/min for layers 68–75. Finally, CD–0–0.9 used a 0.4 mm nozzle, while CD–0–0.4 used a 0.2 mm nozzle.

In addition to previously reported parameters, key critical process parameters (CPPs) were identified and controlled to ensure reproducibility and structural integrity of the CDs. An initial layer height of 0.15 mm was used to promote firm adhesion to the build platform, followed by 0.1 mm layers for the remaining CD. The platform temperature was maintained at 60 °C throughout the printing process to reduce distortion. In terms of cooling, the fan speed was kept at the default value during layers 1 to 63 and deactivated during the final layers (64 to 75) to prevent displacement of the drug powder. These parameters were chosen based on the characteristics of the commercial PVA filament and the functional requirements of the design.

### 2.3. Swelling and Erosion Studies

CDs were immersed in 30 mL of unstirred distilled water at 37 °C and collected after different immersion times (t_im_) to evaluate the water uptake. Additionally, the erosion was studied by placing the swelled CDs in a desiccator until constant weight.

Different t_im_ were considered for each CDs, in particular, as follows:

- t_im_ for CD–0.4 = 0 min, 1 min, and 3 min;
- t_im_ for CD–0.9 = 0 min, 1 min, 5 min, 15 min, and 30 min.

The t_im_ for 0 min implies submerging and immediately withdrawing the CDs from the medium.

After withdrawal, each sample was gently placed on a filter paper for 30 s to remove excess water. Initial weight (*w*_0_) and weight after immersion (*w_im_*) were measured in an analytical balance (Ohaus PA2202, NJ, USA) to calculate the swelling as follows:

(1) $$S\left[\%\right]=\frac{\left(w_{im}-w_{0}\right)}{w_{0}}100$$

Erosion was determined considering the final weight of the CDs after drying (*w_f_*) as follows:

(2) $$E\left[\%\right]=\frac{\left(w_{f}-w_{0}\right)}{w_{0}}100$$

Both studies were performed in triplicate.

### 2.4. Adhesion Test

**Figure 2:**
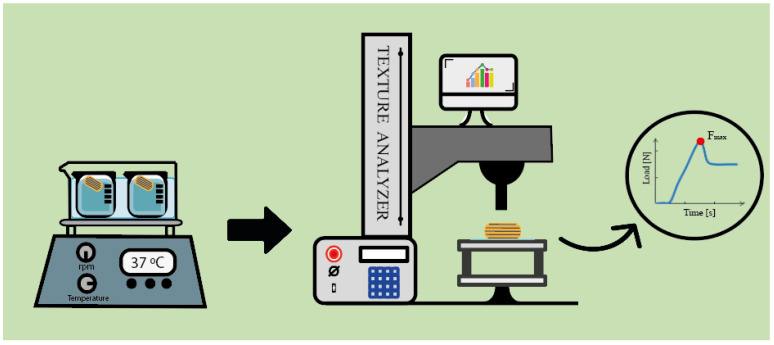
Schematic representation of the experimental setup used for the adhesion test.

A TA Plus texture analyzer (Lloyd Instruments, Bognor Regis, England) equipped with a 5 kg load cell was used to study the adhesion performance of the CDs after being in contact with water. Figure 2 shows the experimental settings for this assay. To this end, the CDs of the swelling test were used. Therefore, the same study conditions and t_im_ were considered. This analysis was performed to characterize the adhesive properties of the CDs and understand the effect of WT, water exposure time, and type of material used on their mechanical behavior.

CD–0–0.9 was exposed to distilled water at 37 °C for 0 (barely wetted), 1, 5, 15, and 30 min, while CD–0–0.4 was exposed for 0, 1, and 3 min. These differences in immersion times are justified by the different opening profiles observed in a disintegration test (see Section 3.3.3) carried out prior to the experiment.

To prevent the CDs from moving during the analysis, they were secured to the base of the apparatus with double-sided adhesive tape. The adhesion test was carried out by applying a cylindrical probe which descended in contact with the CD surface at a speed of 0.1 mm/s, generating a minimum initial contact force. The maximum force (F_max_) in the load vs. time profile required to separate the probe from the CD was then recorded as a measure of the adhesive performance of the material (Figure 2).

To minimize potential experimental errors, maximum force values below 0.5 N were discarded as they were not considered representative of the actual adhesive properties [21]. The assay was done in triplicate.

### 2.5. Water Sorption

Water sorption curves for the PVA filament, HGC, CD–0–0.4, and CD–0–0.9 (empty CDs) were generated to understand the water sorption behavior of the samples and to evaluate the hydrophilic properties of PVA. The HGC was included for comparison with the CDs, while the PVA filament was used to assess the behavior of the material in its pre- and post-printing conditions. The PVA filament samples were sized to weight the same as the empty capsules. Each sample, by triplicate, was stored in closed containers with a glycerine-water solution at 20 °C. These solutions were of a specific concentration to maintain a constant relative humidity (RH) within the container [22]. The samples were weighed periodically until constant weight was achieved. The water sorption at each RH was then calculated.

### 2.6. Differential Scanning Calorimetry (DSC)

DSC was used to evaluate the thermal behavior of LP inside the CDs. The purpose of this analysis was to assess the stability of the drug by monitoring changes in its thermal properties over time. An SDT Q600 instrument (TA Instruments, New Castle, DE, USA) with Universal V4.5A software was used to determine the DSC spectra. Approximately 25 mg of the sample was heated from 30 °C to 300 °C at a rate of 10 °C/min in aluminum crimped pans under a nitrogen gas atmosphere. Instrument calibration was performed using zinc as the standard reference.

### 2.7. Fourier Transform Infrared Spectroscopy (FTIR)

FTIR was used to analyzed LP inside the CDs. The spectra were obtained with a Nicolet 6700 spectrometer, operating in the spectral range 4000–400 cm^−1^ with a resolution of 4 cm^−1^ and average scans of 64. Nitrogen was the purge gas to remove the interference of atmospheric carbon dioxide and water vapor in the sample. A mass of 2 mg of LP was mixed with 198 mg of pure dried KBr, which was used to collect the background spectra.

### 2.8. CDs Weight and Dimensions

Determining the average weight of a 3D-printed capsule is a primary parameter to ensure reproducibility and uniformity of the contents within the CDs. Weight uniformity was assessed in accordance with the International Pharmacopoeia [23] for uncoated tablets, which stipulates that not more than two individual weights should deviate from the average by more than 5%. All the CDs were weighed with an analytical balance (Ohaus PA2202, Parsippany, NJ, USA) and measured with digital caliper gauge (Hamilton Professional Brand, C30, Reno, NV, USA). For both designs (CD–0–0.4 and CD–0–0.9), three samples each were measured.

### 2.9. Disintegration Test

This experiment was conducted in accordance with the procedures established in USP 44-2021 [24] using a disintegration tester apparatus (Scout, Buenos Aires, Argentina). The use of sinkers was necessary to prevent the CDs from floating.

The apparatus was operated at a frequency of 29 to 32 cycles per minute using 800 mL of distilled water at 37 ± 0.5 °C. Results were expressed as mean disintegration time (DT) (s) ± SD. All designs were tested in quadruplicate.

### 2.10. Dissolution Profiles

The in vitro drug dissolution study was performed in a Dissolution Apparatus II (708-DS, Agilent Technologies, Santa Clara, CA, USA) with a paddle rotation of 100 rpm. Distilled water (900 mL) at 37 ± 0.5 °C was used as the dissolution medium. Each 5 mL sample was collected at a predetermined time point (5, 10, 15, 20, 30, 45, 60, 75, 90, and 120 min) and replenished with dissolution medium using a Dissolution Sampling Station (Agilent 8000, Agilent Technologies, Santa Clara, CA, USA) to ensure consistency. The concentration of LP was determined by UV spectrophotometry at 256 nm (UV/VIS Lambda 265 Spectrophotometer PerkinElmer, Waltham, MA, USA). The expression of the calibration curve was y = 0.0185x + 0.0244 with coefficient of determination (R^2^) ≥ 0.998, with a range between 2 and 75 mg/mL.

The experiments were performed in triplicate for CD–0–0.4 and CD–0–0.9 and the mean values are reported. To compare dissolution profiles, the similarity factor *f*_2_ was calculated with the following expression [25]:

(3) $$f_{2}=50\log\left\{100{\left[1+{\left(\frac{1}{n}\sum_{t=1}^{n}{\left(R_{t}-T_{t}\right)}^{2}\right)}^{2}\right]}^{-0.5}\right\}$$

### 2.11. Stability Studies

CDs in triplicate were packaged in airtight plastic containers, sealed, and kept under natural (25 ± 2°C and 60 ± 5% RH) and accelerated conditions for climatic zone II (40 ± 2°C and 75 ± 5% RH reached in a stability chamber-ICH 930L, SCT Pharma, Temperley, Argentina) according to ICH guidelines [26]. The stability of LP inside the CDs was determined by DSC and FTIR at time zero (t_0_) and after one and three months at the two storage conditions. Furthermore, the CDs’ critical quality attributes such as weight, dimensions, disintegration time, and in vitro dissolution were evaluated and statistically compared at all the times and storage conditions.

### 2.12. Statistical Analysis

Data for CD weight and dimensions, disintegration time, and adhesion test were subjected to one-way analysis of variance (ANOVA) at a 5% significance level to assess the presence of statistical differences between the groups analyzed. When significant differences were found, Tukey’s multiple comparison test was used to determine groups exhibiting these differences.

### 2.13. In Vivo Studies

To evaluate the behavior of CDs in the stomach, CDs and HGCs were formulated with BaSO_4_ as a contrast agent for comparison [27]. They were then administered orally to a dog, and radiographs were taken at t_0_, as well as at 2 and 4 min for the HGCs, and at 2, 20, 40, and 50 min for both CDs. Before sample administration, the animal was fasted for 8 h with ad libitum access to water. This study was conducted in accordance with the bioethical protocols of the Catholic University of Cordoba (Argentina, CICUAL Nº012, 10-10-2024) and each case was performed in duplicate.

## 3. Results

### 3.1. Swelling/Erosion and Adhesion Studies

**Figure 3:**
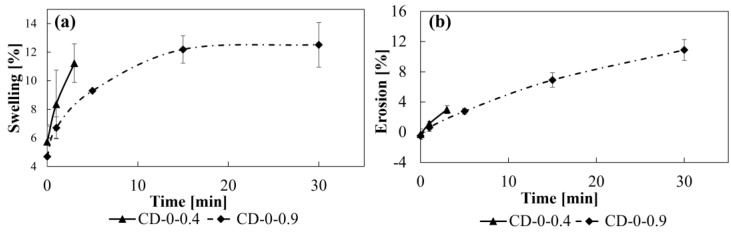
Profiles of (**a**) swelling and (**b**) erosion at different immersion times for CD–0–0.4 and CD–0–0.9.

Adhesion to mucosal surfaces is a critical feature for drug delivery systems and the mucoadhesivity of PVA is influenced by the test conditions [28]. Since swelling capacity and erosion can directly affect adhesion, it is essential to analyze the interaction of both designs with water as a first approach to understanding their performance. As expected, the swelling of the CDs (i.e., water uptake) increases with t_im_ for both designs (Figure 3a). However, the swelling curve of CD–0–0.4 has a steeper slope compared to CD–0–0.9, indicating a higher water uptake rate. This could be attributed to the fact that as WT decreases, the CD structure becomes less resistant to dissolution in water, favoring the formation of micro-cracks that facilitate water penetration and increase swelling. Furthermore, for CD–0–0.9, swelling tends to stabilize at around 20 min, reaching a maximum of 12.5%, corresponding exclusively to material water uptake. This stabilization indicates the formation of a structure more resistant to water ingress, which limits absorption and allows a balance of water retention capacity to be achieved. In relation to erosion (Figure 3b), CD–0–0.4 shows a linear variation with t_im_, slightly faster than for CD–0–0.9, reaching a maximum of 3.8% erosion at 3 min. For CD–0–0.9, erosion also increases linearly with t_im_. For CD–0–0.9, a more gradual and sustained erosion over time is observed, but also with a linear variation. In both cases, there is no stabilization of the erosion values, as is the case for CD–0–0.9 swelling.

**Figure 4:**
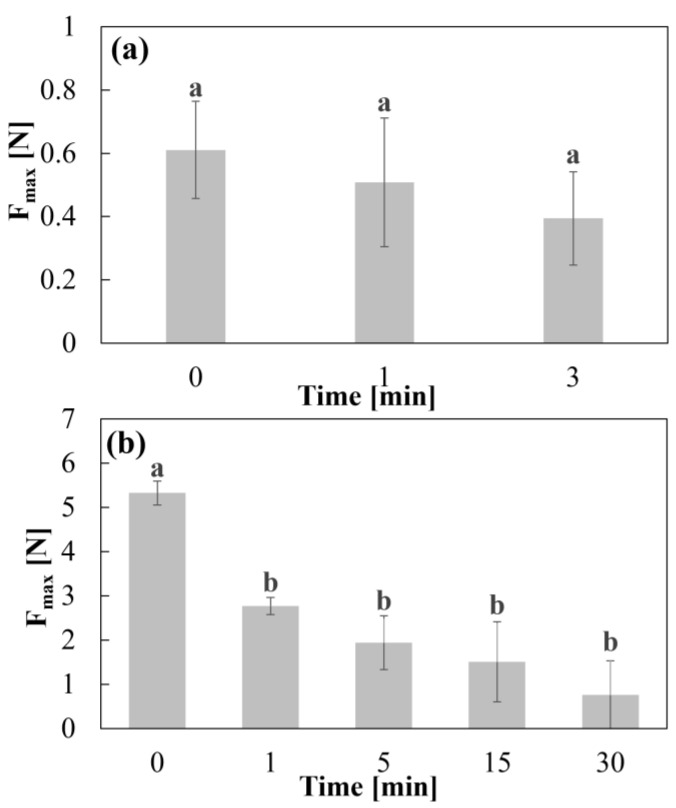
Adhesion test F_max_ values for (**a**) CD–0–0.9 and (**b**) CD–0–0.4. Letters correspond one-way analysis of variance.

For CD–0–0.4, the three t_im_ tested showed no significant differences in adhesion (*p* ≥ 0.05) (Figure 4a). This suggests that at lower WT, water uptake and consequent gelation of the material occurs more rapidly, reaching an equilibrium state where the F_max_ value associated with adhesion remains constant. With respect to CD–0–0.9, it was observed that the higher F_max_ value (5.3 N) occurred at t_0_, with small amounts of water, showing significant differences with respect to the other immersion times (*p* ≤ 0.05) (Figure 4b). Although a decrease in F_max_ values was observed with increasing time, the differences between them were not statistically significant *(p* ≥ 0.05). This reduction in adhesion with respect to t_0_ indicates that water affinity has a strong influence on the mechanical properties of the CDs. Furthermore, the F_max_ values obtained for CD–0–0.9 are nearly one order of magnitude higher than those for CD–0–0.4, indicating that a greater PVA content, associated with a thicker WT, enhances material adhesion.

### 3.2. Water Sorption

**Figure 5:**
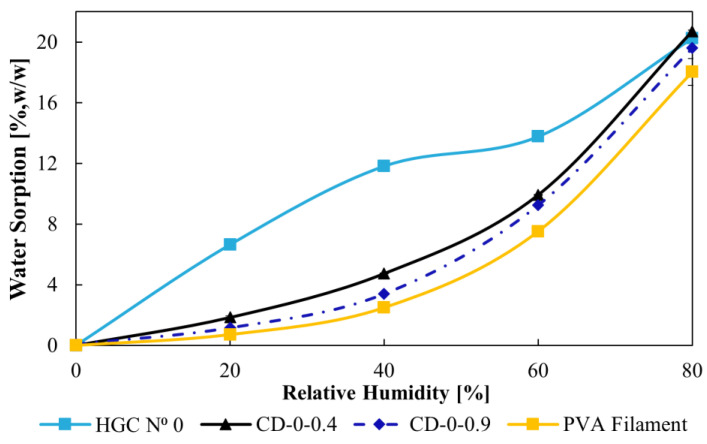
Water sorption as a function of RH for HCG, CDs, and PVA filament.

The results in Figure 5 show the water sorption as a function of RH for HGC, CD–0–0.4, CD–0–0.9, and the PVA filament. The HGC curve shows a distinctly different and significantly higher water sorption profile compared to the other materials over the entire humidity range evaluated except for 80% RH. This difference becomes especially noticeable at 40% RH, where the water sorption of the HGC was considerably higher, while the other designs showed much more moderate values. At this point, the water sorption of HGC was 7.1%, 8.4%, and 9.3% higher than that of CD–0–0.4, CD–0–0.9, and the PVA filament, respectively. After 40% RH, the difference in water sorption between HGC and the other designs remains evident. However, at 80% RH, the sorption values tend to converge due to the saturation of all materials at such a high RH level, which reduces the differences. Despite this, the distinct behavior of HGC across the RH range highlights its high hygroscopicity, with a sharp increase in absorption capacity as RH rises. In contrast, CDs and PVA filament showed a more controlled and gradual absorption pattern, suggesting a lower affinity for moisture and enhanced stability under moderate conditions.

### 3.3. Stability Studies

#### 3.3.1. Drug DSC and FTIR

**Figure 6:**
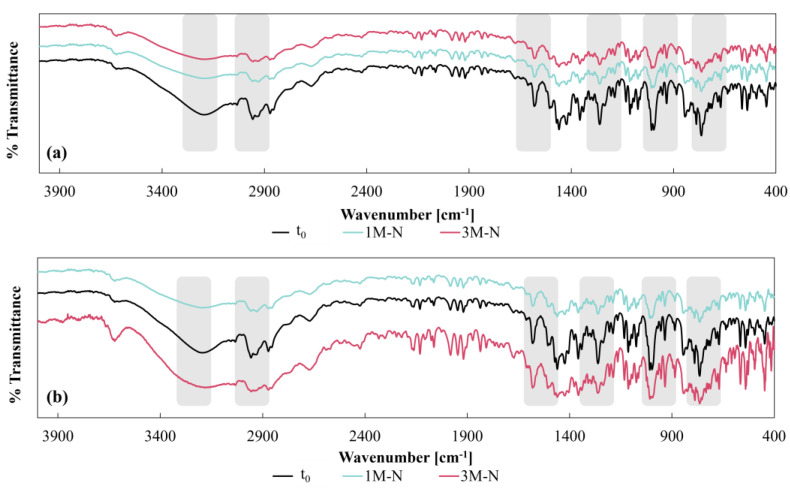
FTIR spectra for LP. (**a**) CD–0–0.4 and (**b**) CD–0–0.9 at t_0_, and one and three months under natural conditions.

The FTIR spectra obtained for the samples stored under natural conditions (1 and 3 months, 1M-N and 3M-N, respectively) showed remarkable agreement with the t_0_ spectrum of LP testing both CDs designs (Figure 6a,b). In fact, the main characteristic peaks of LP remained unchanged. In particular, the band around 764 cm^−1^ was observed, associated with C-Cl bond stretching vibrations; C-O stretching vibrations and aromatic ring breathing were recorded in the region from 997 to 1074 cm^−1^; the band at 1260 cm^−1^ is attributed to C-N stretching vibrations; the absorption bands around 1580 cm^−1^ are associated with N=N and C=C stretching vibrations; the band at 2956 cm^−1^ is associated with asymmetric -CH_3_ vibrations; and the broad band around 3190 cm^−1^ is associated with stretching of the LP-OH group. The persistence of these bands, without significant changes in their position or intensity, confirms the chemical stability of LP inside the CDs under natural conditions during the period studied. The spectra obtained are in agreement with previous reports [29] which describe these peaks as characteristic of the polymorphic form I of LP.

**Figure 7:**
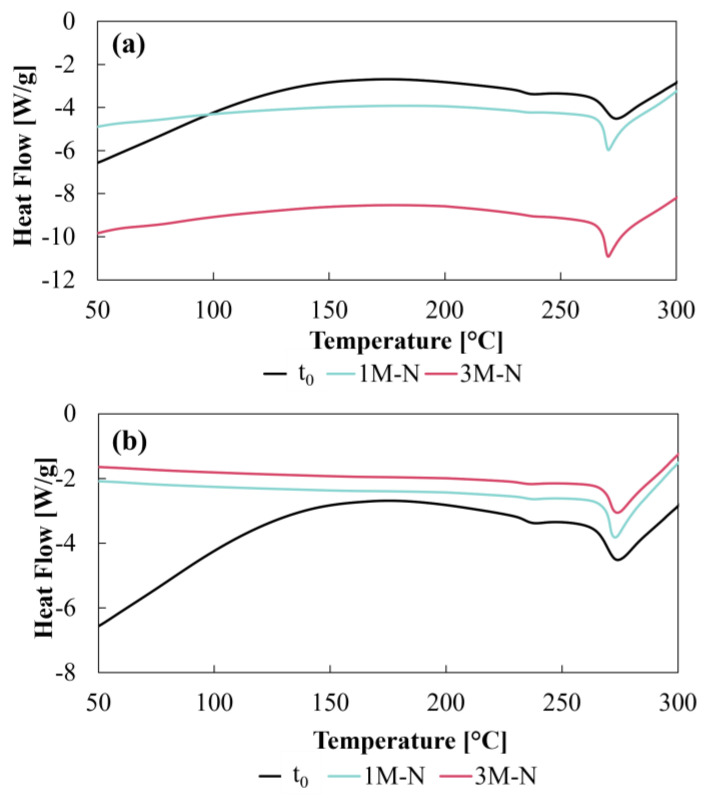
DSC thermograms for LP. (**a**) CD–0–0.4 and (**b**) CD–0–0.9 at t_0_, and one and three months under natural conditions.

The thermogram of the LP at t_0_ showed a characteristic thermal behavior of the drug, consistent with that reported in the literature [29]. For CD–0–0.4 (Figure 7a), a small endothermic peak was observed at about 238.63 °C, associated with an enantiotropic polymorphic transition from form I to form II during heating. In addition, a larger endothermic peak was observed at 274.07 °C, corresponding to the melting point of the drug. These values agree with those reported for the polymorphic form I of LP [29]. Thermograms of samples stored under 1M-N and 3M-N showed thermal profiles comparable to those of LP at t_0_. The endothermic peaks for CD–0–0.9 (Figure 7b) corresponding to the polymorphic transition and melting point were maintained at similar temperatures, with values of 237.93 °C and 272.87 °C for the 1M-N samples and 235.94 °C and 273.8 °C for the 3M-N samples, respectively. For CD–0–0.4-stored 1M-N, the above two peaks occurred at 238.75 °C and 275.08 °C and for 3MN at 238.09 °C and 271.04 °C. The absence of significant changes in transition and melting temperatures, as well as the preservation of peak shape, indicates that there were no changes in the crystal structure of LP during storage under natural conditions.

Conversely, the accelerated stability conditions revealed limitations in the physical integrity of the CDs. After one month of exposure, the excessive moisture uptake and deep impregnation of LP into the PVA matrix hindered the acquisition of reliable FTIR spectra and DSC thermograms. Moreover, after three months under these conditions, structural compromise was evident, as the CDs were found to be open, which prevents further physicochemical analysis.

#### 3.3.2. CDs Weight and Dimensions

**Figure 8:**
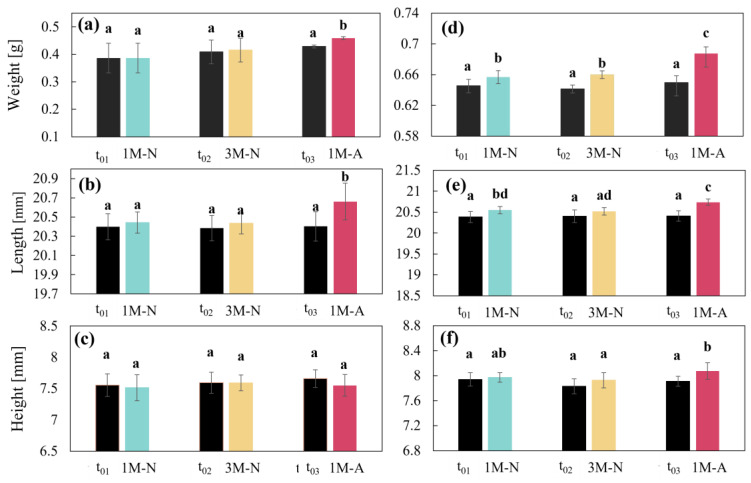
(**a**) Weight, (**b**) length, (**c**) height for CD–0–0.4 in different storage conditions, and (**d**) weight, (**e**) length, and (**f**) height for CD–0–0.9 in different storage conditions. Letters correspond one-way analysis of variance.

The results showed differences in weight and dimensions of the two designs. CD–0–0.4 showed greater dimensional stability. Only weight and length (Figure 8a,b) were significantly affected by the accelerated conditions at 1 month (1M-A) (*p* < 0.05). This indicates that CD–0–0.4 kept its dimensions (height, length) (Figure 8b,c) and weight under natural conditions relatively constant, except for an increase in weight and length under accelerated conditions at 1 month.

In contrast, CD–0–0.9 showed a significant increase in weight (Figure 8d) compared to t_0_ under all storage conditions. Tukey’s post-hoc analysis confirmed these differences and showed that the 1M-N, 3M-N, and 1M-A groups were significantly different from t_0_ (*p* < 0.05). This increase in weight is attributed to the higher water uptake capacity of PVA, which is more prevalent in CD–0–0.9 due to the presence of more material to hydrate. Similar to weight, length (Figure 8e) was also significantly affected by all storage conditions. For height (Figure 8f), CDs stored for 1M-A showed a significant effect, confirmed by Tukey’s test.

#### 3.3.3. Disintegration Test and Dissolution Profiles

Table 1 shows the values of DT obtained for CD–0–0.4 and CD–0–0.9. It is possible to observe an increase in the DT with increasing WT. For CD–0–0.4, ANOVA test showed no significant differences between the different storage conditions, with values ranging from 437 s to 524 s, indicating that the CDs maintained their structural integrity regardless of storage conditions.

In contrast, for CD–0–0.9, the statistical analysis showed significant differences between the storage conditions. At t_0_, CD–0–0.9 demonstrated the longest DT (1510 s), whereas samples stored at 1M-N and 3M-N storage conditions exhibited progressively shorter times of 1139 s and 1050 s, respectively. The most pronounced reduction was observed under 1M-A condition (i.e., 741 s), which exhibited statistically significant differences compared to the other storage conditions, as confirmed by the Tukey’s test.

**Figure 9:**
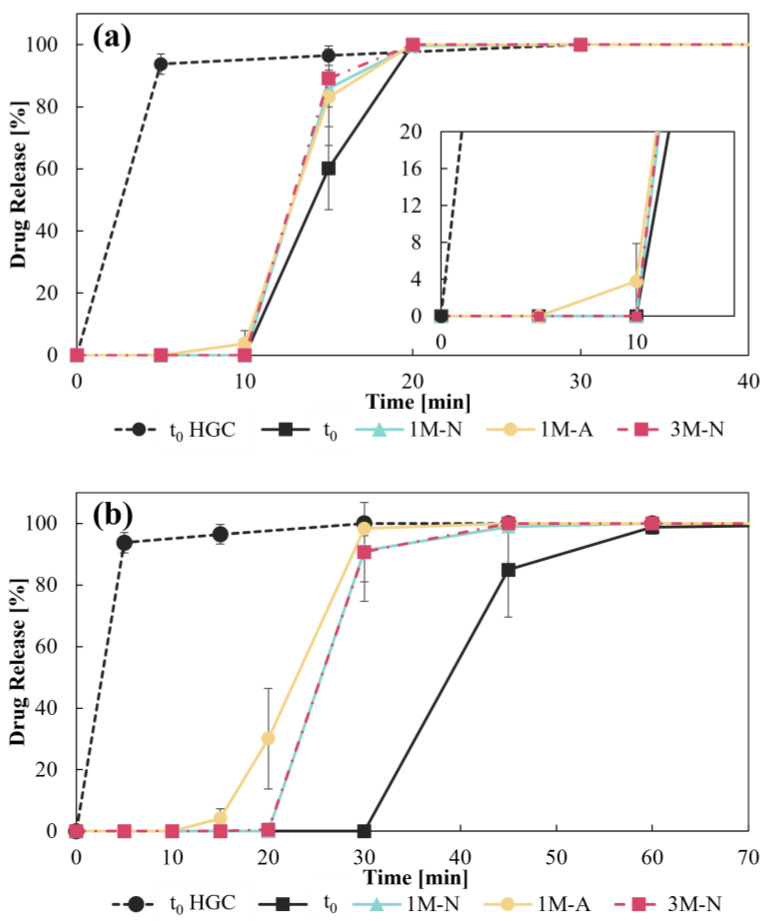
Dissolution test for (**a**) CD–0–0.4 and (**b**) CD–0–0.9 at t_0_ and different storage conditions.

For CD–0–0.4, as shown in Figure 9a, the dissolution profiles did not exhibit any significant differences across the various storage conditions.

Dissolution analyses were performed on both CDs designs stored at different times and under different conditions. Figure 9 shows the HGC release profile at t_0_ as reference of an immediate drug-release behavior. For CD–0–0.4, as shown in Figure 9a, the dissolution profiles did not exhibit any significant differences for the tested storage conditions. This stability is consistent with the DT results, where no significant variations in the opening time of CDs were observed. The values of *f_2_* greater than 50 (Table 2) further confirm this behavior, which is beneficial for maintaining the integrity of the CDs. The release of the drug initiates after 10 min (with the exception of 1M-A) and achieves complete release (100%) within 20 min.

Consistent with the results of DT, the release profiles of the drug from CD–0–0.9 (Figure 9b) showed significant variability as a function of storage conditions. The *f_2_* values from the comparison of t_0_ with the storage conditions were significantly lower than 50, indicating notable differences in the profiles. Nevertheless, pairwise comparisons of release profiles at different storage conditions, such as 1M-N/1M-A, 3M-N/1M-A, and 3M-N/1M-A, showed similarities (*f*_2_ > 50) (Table 2). At t_0_, drug release starts at 30 min and reaches 100% at 60 min. On the other hand, the release profiles under different storage conditions showed a shift to the left of the abscissa axis, with drug release starting at shorter times (i.e., 20 min for 1M-N and 3M-N) and reaching complete release at 45 min. For the 1M-A condition, the release is more gradual, starting at 15 min and reaching 100% at 30 min. The shift to the left of the dissolution profiles coincides with the progressive reduction in DT, and these times align with the initial drug release. Based on these results, it can be stated that storage conditions affect the release of the drug from CD–0–0.9, but not from CD–0–0.4. This difference can be attributed to the mechanical properties of each design, which are influenced by their WT. CD–0–0.4 is at a structural threshold, enabling rapid and immediate opening upon exposure to the medium, regardless of storage conditions. In contrast, CD–0–0.9, with its more robust structure, may exhibit greater sensitivity to variations in storage conditions, leading to performance similar to CD–0–0.4, which affects its opening and subsequent dissolution. To summarize, both HGC-0 and CD–0–0.4 released more than 80% of the drug within the first 30 min, corresponding to an immediate release dosage form. In contrast, CD–0–0.9 showed a slower profile under initial conditions, reaching 80% drug release at around 60 min, indicating a delayed release behavior. However, under accelerated conditions, CD–0–0.9 showed a faster release, with 80% of the drug being released within 30–45 min, approaching an immediate release profile [30].

### 3.4. In Vivo Studies

**Figure 10:**
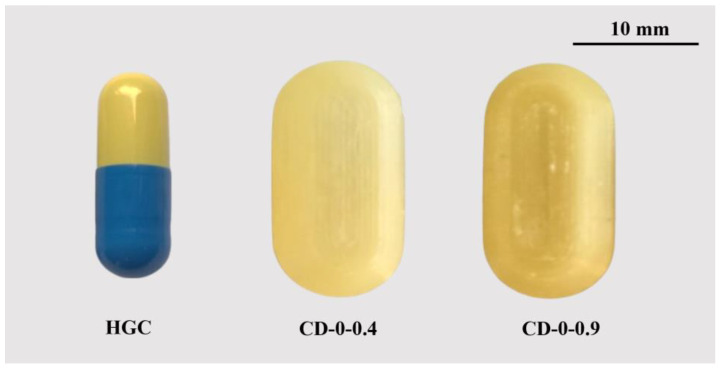
HGC size 0 and CDs used for the in vitro and in vivo studies.

To provide a clear reference for the capsules analyzed in this study, Figure 10 illustrates the actual appearance of HGC, CD–0–0.4, and CD–0–0.9 right after the printing process.

**Figure 11:**
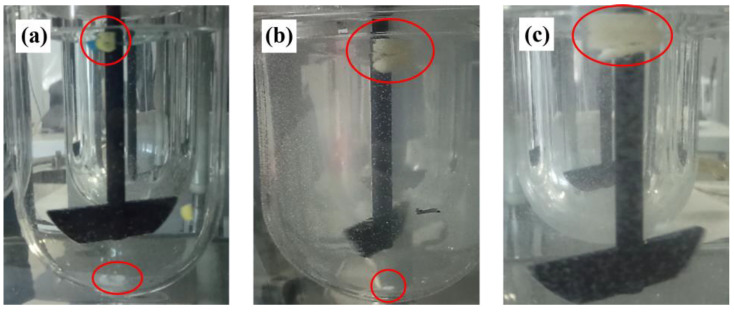
Opening of (**a**) HGC size 0, (**b**) CD–0–0.4, and (**c**) CD–0–0.9 in the in vitro study. The red circles highlight the location of the contrast agent.

In the first place, a dissolution test of the capsules filled with BaSO_4_ (X-ray contrast agent) was designed to later correlate with the in vivo studies (see Section 2.12). The dissolution apparatus described in Section 2.8 was used. Both HGC and CDs were tested without sinkers. Opening times were registered when cracks or BaSO_4_ release into the medium were observed. The opening times were 17 ± 5 s, 12 ± 3 min, and 33 ± 3 min for HGC, CD–0–0.4, and CD–0–0.9, respectively. Figure 11 illustrates the opening of the designs. Due to the low solubility of the contrast agent, it primarily remained within the CD structure, while a small portion precipitated at the bottom of the vessel. For the HGC, the structure was completely lost, releasing all the contrast agent as soon as it opened.

**Figure 12:**
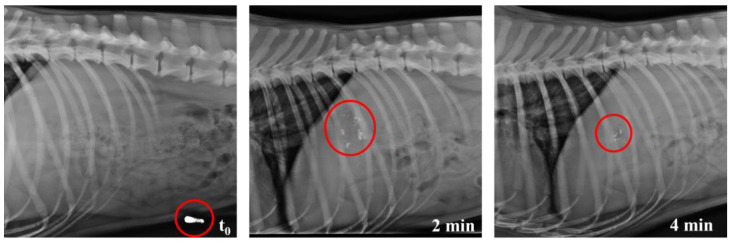
X-ray images of dog’s abdomen showing the HGCs filled with BaSO_4_ at different time points.

**Figure 13:**
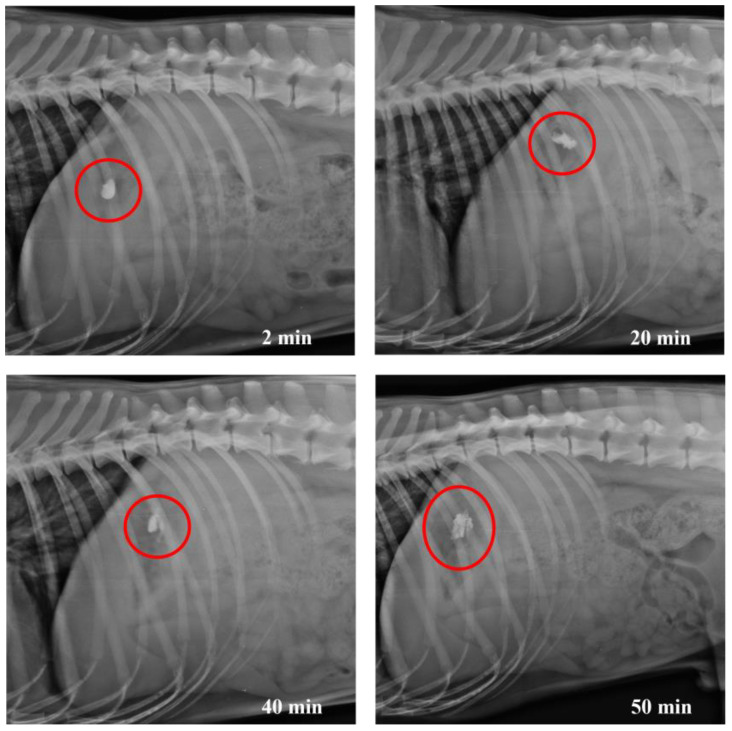
X-ray images of dog’s abdomen showing the CD–0–0.4 filled with BaSO_4_ at different time points.

**Figure 14:**
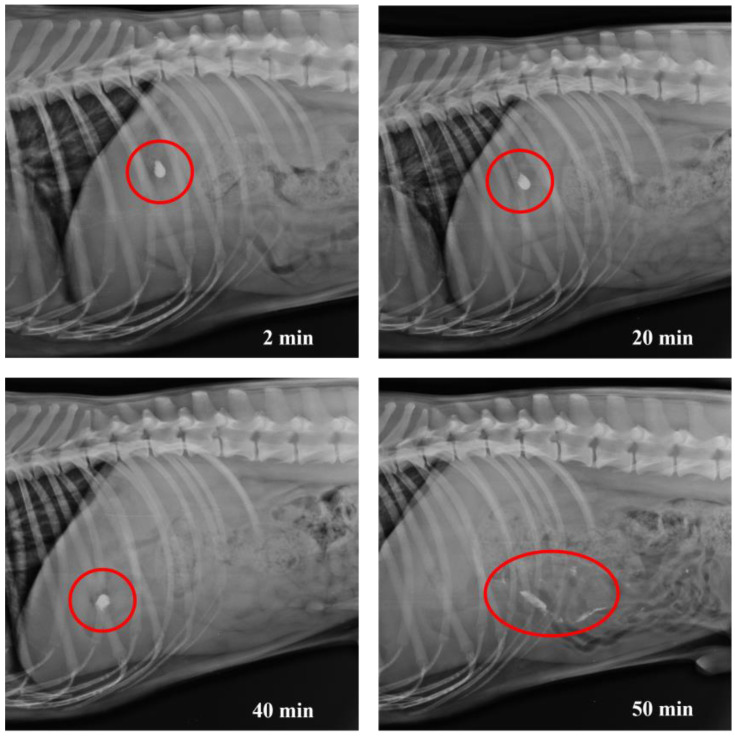
X-ray images of dog’s abdomen showing the CD–0–0.9 filled with BaSO_4_ at different time points.

X-ray images taken at different times show the evolution of the opening of HGCs (Figure 12) as well as CD–0–0.4 (Figure 13) and CD–0–0.9 (Figure 14). As previously observed in the in vitro studies, the opening of the designs can be inferred when the structure is slightly compromised due to the partial release of BaSO_4_. The rest of the contrast agent remains inside the designs. For HGCs, it was observed that after 2 min, the capsule had reached the stomach and disintegrated immediately. By 4 min, the HGC was found to be fully disintegrated. This phenomenon is consistent with the in vitro results and the composition of the capsule, which is formulated for immediate release into the gastric environment. For CDs, opening times varied with thickness. In vivo results showed CD–0–0.9 kept its shape until around 40 min before showing signs of opening. Conversely, CD–0–0.4 began to open and deform after 20 min, indicating that the capsule’s mechanical strength is crucial for its stability and opening kinetics. These results align with the in vitro opening times, indicating that the proposed in vitro study is appropriate for predicting the behavior of these CDs under physiological conditions.

## 4. Conclusions

The present study evaluated the in vivo performance and stability of PVA-CDs with different WTs (i.e., 0.4 mm and 0.9 mm) compared to HGC. In vivo opening times of HGC and CDs filled with a contrast agent were assessed in dogs using X-ray images. HGC reached the stomach within 2 min and disintegrated immediately, which is typical for this type of capsule. CDs’ opening times varied with WT, with CD–0–0.4 opening the fastest. Additionally, the in vivo opening times correlated well with ad hoc design in vitro assay, where a dissolution apparatus was used to evaluate capsule opening. CD–0–0.4 showed higher swelling, erosion, lower adhesion, faster disintegration, and quicker drug release than CD–0–0.9. Stability tests demonstrated that the drug chemical structure, weight, dimensions, disintegration time, and drug dissolution profile of CD–0–0.4 were not affected by the storage conditions. Finally, the water sorption test indicated a lower moisture affinity of PVA capsules compared to HGC. These comprehensive in vivo and in vitro evaluations of the CDs suggest that CD–0–0.4 offers immediate drug release and appropriate stability, making it a suitable option for use in magistral compounding pharmacy applications.

## Figures and Tables

**Table 1:** Disintegration time for both CDs designs at different storage conditions.

	Disintegration Time [s]	
	CD–0–0.4	CD–0–0.9
t_0_	524 ± 25	1510 ± 54
1M-N	509 ± 5	1139 ± 89
3M-N	524 ± 38	1050 ± 54
1M-A	437 ± 24	741 ± 161

**Table 2:** *f*_2_ values between the dissolution profiles from CD–0–0.4 and CD–0–0.9 at different storage conditions.

	CD–0–0.4			CD–0–0.9		
	1M-N	1M-A	3M-N	1M-N	1M-A	3M-N
t_0_	54.2	56.5	51.8	26.7	24.1	26.8
1M-N		92.6	89.3		50.2	98.4
1M-A			80.4			50.4

## Data Availability

Data are contained within the article.
